# Improving phosphorus acquisition efficiency through modification of root growth responses to phosphate starvation in legumes

**DOI:** 10.3389/fpls.2023.1094157

**Published:** 2023-02-10

**Authors:** Zhijian Chen, Linjie Wang, Juan Andres Cardoso, Shengnan Zhu, Guodao Liu, Idupulapati M. Rao, Yan Lin

**Affiliations:** ^1^ Key Laboratory of Tropical Crops Germplasm Resources Genetic Improvement and Innovation of Hainan Province, Institute of Tropical Crop Genetic Resources, Chinese Academy of Tropical Agricultural Sciences, Haikou, China; ^2^ International Center for Tropical Agriculture (CIAT), Cali, Colombia; ^3^ Life Science and Technology School, Lingnan Normal University, Zhanjiang, China; ^4^ International Centre of Insect Physiology and Ecology (icipe), Nairobi, Kenya; ^5^ Institute of Bioengineering, Guangdong Academy of Sciences, Guangzhou, China

**Keywords:** phosphorus deficiency, root morphology, root architecture, root traits, Pi uptake, PSI genes

## Abstract

Phosphorus (P) is one of the essential macronutrients for plant growth and development, and it is an integral part of the major organic components, including nucleic acids, proteins and phospholipids. Although total P is abundant in most soils, a large amount of P is not easily absorbed by plants. Inorganic phosphate (Pi) is the plant-available P, which is generally immobile and of low availability in soils. Hence, Pi starvation is a major constraint limiting plant growth and productivity. Enhancing plant P efficiency can be achieved by improving P acquisition efficiency (PAE) through modification of morpho-physiological and biochemical alteration in root traits that enable greater acquisition of external Pi from soils. Major advances have been made to dissect the mechanisms underlying plant adaptation to P deficiency, especially for legumes, which are considered important dietary sources for humans and livestock. This review aims to describe how legume root growth responds to Pi starvation, such as changes in the growth of primary root, lateral roots, root hairs and cluster roots. In particular, it summarizes the various strategies of legumes to confront P deficiency by regulating root traits that contribute towards improving PAE. Within these complex responses, a large number of Pi starvation-induced (PSI) genes and regulators involved in the developmental and biochemical alteration of root traits are highlighted. The involvement of key functional genes and regulators in remodeling root traits provides new opportunities for developing legume varieties with maximum PAE needed for regenerative agriculture.

## 1 Introduction

Phosphorus (P) is one of the principal macronutrients for plant growth and productivity, and it is part of the crucial organic components such as nucleic acids, proteins, enzymes and phospholipids (Hawkesford et al., 2012; Plaxton and Shane, 2015; Lambers, 2022). P participates in a series of physiological, biochemical and metabolomic processes in plants such as photosynthesis, respiration, energy generation, nucleic acid synthesis, nitrogen (N) fixation and redox reactions (Liang et al., 2014; Ham et al., 2018; Lambers, 2022). Hence, P is essential at all developmental stages of plants including seed germination, root growth, leaf and stem development as well as flower and seed generation (Malhotra et al., 2018).

Although total P is abundant in most soils, a large proportion of P is fixed by soil mineral components (e.g., aluminium or iron) into insoluble chemical complexes that are not readily accessible to plants (Hinsinger, 2001; Margenot et al., 2016; Ojeda-Rivera et al., 2022). Inorganic phosphate (Pi), in the form of HPO_4_^2-^ and H_2_PO_4_^-^, is the plant- available P. However, Pi concentration in soil solutions is generally less than 10 µM (Vance et al., 2003; Mo et al., 2022). Therefore, low Pi availability is considered as a major limiting factor for plant growth, development and yield in more than 60% of the world’s arable land (Gutiérrez-Alanís et al., 2018). To obtain high crop yields, a large amount of P-containing fertilizers derived from rock phosphate are applied in agricultural systems. Approximately 60 million tonnes of P fertilizers were used in 2020 around the world, which were more than 40% higher than those in 2000 (FAOSTAT, 2022). However, only 10–30% of the P in P fertilizers are estimated to be used by plants (Vance et al., 2003; Richardson and Simpson, 2011; Ojeda-Rivera et al., 2022). Most of the mineral P fertilizers that are applied in high amounts can gradually leach from soils into water bodies, leading to environmental pollution, such as eutrophication (MacDonald et al., 2011; Zak et al., 2018). In addition, rock phosphate reserves are a non-renewable resource that will be depleted in future (Vance et al., 2003; George et al., 2016). Breeding programs address this problem through the development of P-efficient crop cultivars that produce higher yields per unit of P fertilizer input. Therefore, an improved understanding of the mechanisms of P efficiency in crops is required.

Several studies have demonstrated that increasing plant P efficiency can be achieved by improving P acquisition efficiency (PAE) and/or P utilization efficiency (PUE) (Wang et al., 2010; Adem et al., 2020; Han et al., 2022; Zou et al., 2022). PAE is regarded as the ability of plants to acquire soil P by roots, while PUE is thought to be the ability of plants to generate biomass or yield using the acquired P (Wang et al., 2010; Han et al., 2022). In P-limited soils, enhancement of PAE is a key strategy that has received considerable attention with a focus on optimizing root traits including: (1) root growth responses that involve changes in root morphology (e.g., primary root, lateral roots, root hairs, and cluster roots) and root architecture, contributing to acquire more P from soils by extension of root system (Lynch, 2011; Li et al., 2016; Zhang et al., 2018; Chen et al., 2021; Liu, 2021; Lynch et al., 2022); (2) coordination of physiological and biochemical alterations of root traits, such as exudation of protons, organic acids and phosphatases into the rhizosphere, facilitating P mobilization from the unavailable P in the rhizosphere (Pang et al., 2018a; Robles-Aguilar et al., 2019; Wen et al., 2019); (3) establishing symbiotic interactions with beneficial microbes (e.g., Pi-solubilizing bacteria) or arbuscular mycorrhizal fungi (AMF) to improve PAE by solubilizing and foraging P (Khan et al., 2014; Campos et al., 2018). Thus, genetic modification of root system traits can be an effective strategy for improving crop varieties with low P tolerance and high PAE.

The Fabaceae family, formerly known as Leguminosae, is one of the largest families of flowering plants, comprising more than 700 genera and about 18,000 species among the grain, pasture, and agroforestry species, and it is second in importance to human activities after Gramineae family (Lewis et al., 2005; Abdelrahman et al., 2018). Legumes account for approximately 27% of the world’s crop production, ranking in second place as human food crops after cereal crops. Major legume crops include soybean (*Glycine max*), common bean (*Phaseolus vulgaris*), chickpea (*Cicer arietinum*), cowpea (*Vigna unguiculata*), pigeon pea (*Cajanus cajan*), groundnut (*Arachis hypogaea*), and white lupin (*Lupinus albus*). Alfalfa (*Medicago sativa*), clover (*Trifolium* spp.), and stylo (*Stylosanthes* spp.) are major forage legumes in the world. Many legume crops are either used for food or as an animal fodder or for both purposes (Foyer et al., 2016; Abdelrahman et al., 2018; Roy et al., 2020). Unlike most other non-legume plants, legumes can develop symbiotic interaction with rhizobia to form nodules that can fix atmospheric N, thereby contributing to enhance agricultural sustainability (Abdelrahman et al., 2018; Yang et al., 2022). Since symbiotic N fixation (SNF) in nodules requires significant inputs of energy, legumes are generally considered to have a high P requirement (Sulieman and Tran, 2015; Pang et al., 2018b; Zhong et al., 2023). Furthermore, as N-fixing root nodules are strong P sinks, the growth and yield of legumes are dramatically decreased by 30–40% under low P stress (Tesfaye et al., 2007; Valdés-López and Hernández, 2008; Chen et al., 2011; Qin et al., 2012; Guo et al., 2022). Thus, low P availability is regarded as an important constraint for legume production.

Over the past three decades, great efforts have been made to elucidate the plant responses and the adaptive mechanisms of legumes to Pi deprivation. In this review, we focus on progress in the understanding of root growth responses to P deficiency in legumes. In particular, we summarize the recent advances in dissecting the adaptive strategies of legume plants to Pi starvation through regulation of root response for improving PAE. We highlight the Pi starvation-induced (PSI) genes that have been successfully characterized for their roles in improving PAE and we also indicate future research directions for improving PAE. This will provide new opportunities for developing legume varieties with high P efficiency that are needed for resource-efficient and regenerative agriculture.

## 2 Legumes adapt to Pi starvation by regulating root growth responses

**Figure 1:**
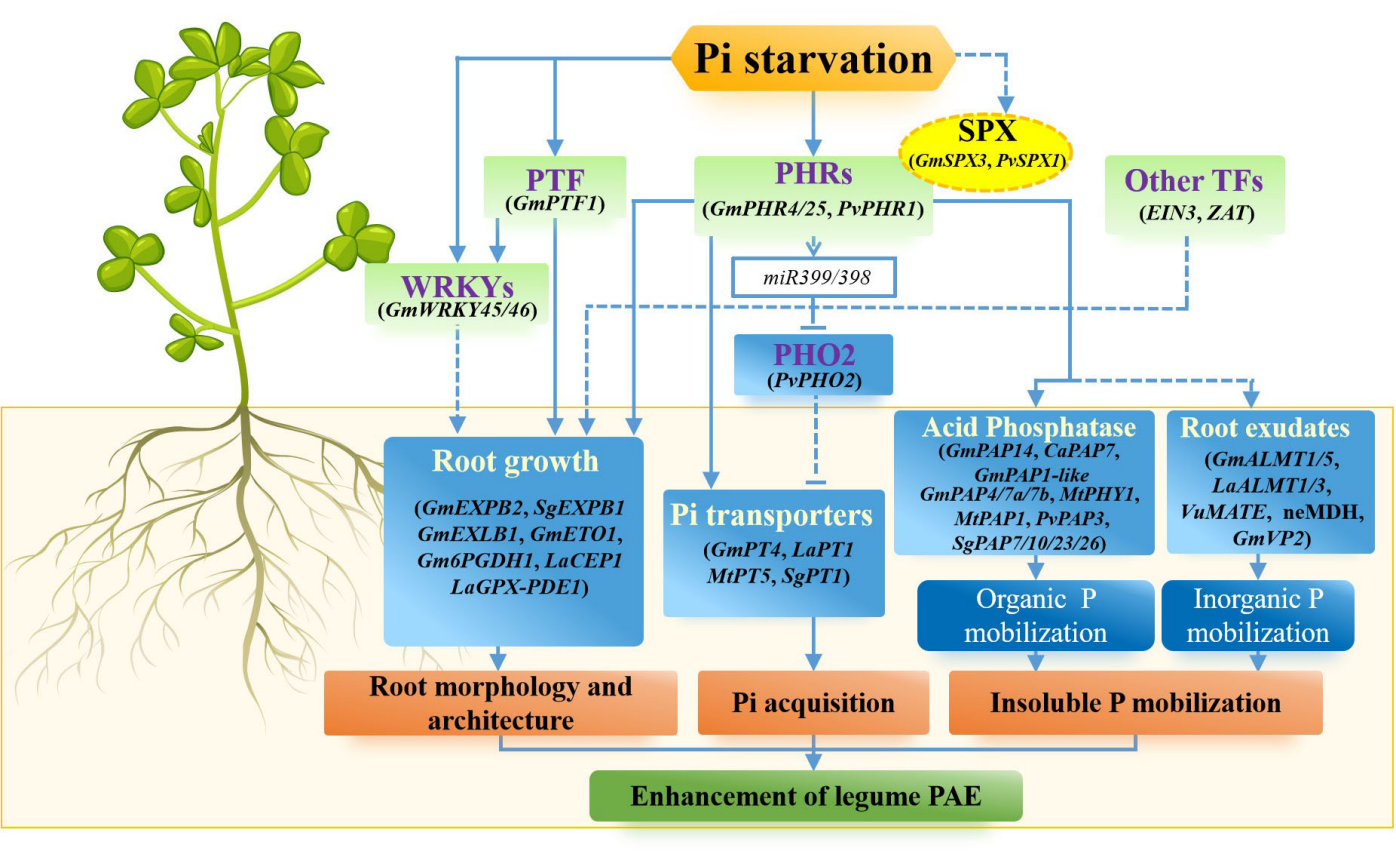
Strategies for improving P acquisition efficiency through gene regulation in legumes. In legumes, P acquisition efficiency can be achieved by remodeling of root morphology and architecture, inducing high-affinity Pi transporters, increasing root exudates to facilitating P mobilization, and activating Pi signaling network. A variety of Pi starvation-induced (PSI) genes have been implicated in improving P acquisition efficiency in legumes. These are related to root growth, Pi uptake, insoluble P mobilization and Pi signaling network.

Plants can sense external and internal Pi status and remodel root traits in response to P deficiency through local and systemic responses (Raya-González et al., 2021). Local P deficiency appears to be the external driver of primary root growth inhibition, promoting lateral root formation and increasing the production of root hairs (Chien et al., 2018; Huang and Zhang, 2020; Liu, 2021) (**Figure 1**). Although the dissection of root responses to P deficiency in legumes has remained less defined, legumes are able to adapt to P deficiency through regulation of a variety of root growth responses and gene expressions.

### 2.1 Root morphology

Adaptive responses of legume plants to P deficiency involve changes in root morphology induced by dynamic remodeling of primary roots, lateral roots, root hairs and cluster roots, which maximize the acquisition of external Pi from soils. Low-P-enhanced elongation of primary roots is observed in many legume plants, such as soybean, common bean, stylo and crowtoe (*Lotus corniculatus*) (Wissuwa, 2003; Guo et al., 2011; Zhou et al., 2014; Luo et al., 2020; An et al., 2023). For example, the growth of primary root is stimulated in soybean and stylo during P deficiency, which may be beneficial for foraging P from soils (Li et al., 2015; Luo et al., 2020; Yang et al., 2021a; Xie et al., 2022). To date, a set of PSI genes have been demonstrated to be involved in the regulation of primary root growth in legumes (Guo et al., 2011; Li et al., 2022). It has been reported that more than 200 PSI genes have been identified in soybean roots. Among them, *GmEXPB2*, a β-expansin gene, is found to be induced by P deficiency in roots. GmEXPB2 is a secretory protein that localizes to the cell wall; overexpression of *GmEXPB2* increases the growth of primary roots by enhancing the size and number of cortical cells in both the root meristematic and elongation zones, thereby increasing Pi uptake and P efficiency in soybean (Guo et al., 2008; Guo et al., 2011; Zhou et al., 2014; Li et al., 2015; Yang et al., 2021a). Furthermore, a low-P-induced expansin-like B gene, *GmEXLB1*, has been proved to participate in enhancing root elongation and modifying root architecture, which is contributed to increase plant PAE (Kong et al., 2019). Similar role of *SgEXPB1* gene in regulating root growth has recently been characterized in stylo under P-deficient condition (Wang et al., 2023). A variety of expansin genes upregulated by P deficiency are also reported in other legumes, such as alfalfa (Li et al., 2022), suggesting the key role of expansin genes in legume root growth. In addition, a group of transcription factors are also reported to be involved in regulating the primary root growth during P deficiency, such as members belonging to the MYB transcription factor family. For example, GmWRKY46, belonging to the WRKY family localized in the nucleus, is induced by P deficiency in soybean roots; overexpression of *GmWRKY46* promotes the growth of both primary root and lateral roots and increases Pi uptake in transgenic Arabidopsis probably through the regulation of downstream PSI gene (Li et al., 2021).

Lateral roots also play vital roles in efficient Pi acquisition by enhancing soil exploration (Li et al., 2020; Zhang et al., 2020; Li et al., 2021; Lynch, 2022). It has been shown that the growth and proliferation of lateral roots in legume plants are mediated by Pi availability. For example, elongation and density of lateral roots are increased by low P stress in alfalfa and common bean (Williamson et al., 2001; Linkohr et al., 2002; Reymond et al., 2006; Zhang et al., 2014). A group of genes involved in lateral root growth have been identified in alfalfa, soybean and white lupin (Cheng et al., 2011; Li et al., 2020; Zhang et al., 2020; Li et al., 2021). For example, *Gm6PGDH1*, encoding the 6-phosphogluconate dehydrogenase, is mainly expressed in the P-deficient soybean root; overexpression of *Gm6PGDH1* increased lateral root length and Pi uptake in transgenic soybean plants under P-deficient conditions (Li et al., 2021). In addition, GmETO1 is an essential ethylene-biosynthesis regulator located in the cell nucleus; both hairy root length and number of lateral roots are significantly increased in transgenic soybean plants with *GmETO1* overexpression (Zhang et al., 2020). Furthermore, it has been found that the overexpression of *GmWRKY45* increases the adaptability of transgenic Arabidopsis to Pi starvation through an increase in lateral root growth, contributing to greater Pi uptake (Li et al., 2020). In white lupin, the NAC domain-containing LaNAC1 is also implicated in regulating the growth of lateral roots under P deficiency (Cheng et al., 2011).

Root hairs, deriving from root epidermal cells, increase the root surface area that can be in contact with the soil substrate (Williamson et al., 2001; Huang et al., 2017). For instance, the number and length of root hairs in alfalfa increase rapidly in the early stage of P deficiency, facilitating greater Pi uptake (Li et al., 2018). Many genes have been documented to be involved in root hair growth in common bean, soybean and white lupin (Cheng et al., 2011; Yao et al., 2014a; Yao et al., 2014b; Li et al., 2015). For example, two genes encoding glycerophosphodiester phosphodiesterase, *LaGPX-PDE1/2*, have been implicated in root hair growth and development in white lupin (Cheng et al., 2011). Both *LaGPX-PDE1/2* are highly expressed in P-limited root hairs; knockdown of *LaGPX-PDE1/2* in white lupin impairs root hair development and density, thereby decreasing P concentration (Cheng et al., 2011). Therefore, *LaGPX-PDE1/2* are proposed to be involved in improving PAE by enhancing root hair development. In common bean, PvSPX1 is one of the SPX (SYG1, Pho81 and XPR1) domain-containing proteins that is localized in the nucleus and plays a central role in the P signaling network (Yao et al., 2014b). The expression of *PvSPX1* is enhanced by P deficiency in both leaves and roots of common bean; overexpression of *PvSPX1* leads to an increase root P concentration and an enlargement of root hair zone in transgenic bean hairy roots, suggesting that PvSPX1 can regulate the growth of root hairs (Yao et al., 2014b).

Cluster roots, also known as proteoid roots, are a specialized root structure consisting of closely spaced tertiary lateral roots, and it is the feature of the *Proteaceae* members and several other plant species (Shane and Lambers, 2005; Shu et al., 2005; Lambers et al., 2006). It has been established that Pi acquisition capacity within the cluster roots is greater than that of the normal roots, suggesting an important role for cluster roots in Pi acquisition (Keerthisinghe et al., 1998). White lupin is the representative plant used to study the formation and growth of cluster roots affected by P nutrition (Liu et al., 2005; Wang et al., 2015). The earliest response of white lupin to P deficiency is the formation of cluster roots (Neumann et al., 2000). Numerous PSI genes have been identified in cluster roots of white lupin (Zhou et al., 2019). Among them, a novel C terminally encoded peptide gene *LaCEP1* is characterized to be negatively regulating cluster root development (Zhou et al., 2019). *LaCEP1* is highly expressed in the pre-emergence zone of the cluster roots; overexpression of *LaCEP1* results in the inhibition of cluster root formation (Zhou et al., 2019). Although the formation of cluster roots significantly increases root surface area, cluster roots increase P acquisition mainly through increasing root exudation rather than by strengthening P foraging (Gardner et al., 1982; Neumann and Römheld, 1999; Shen et al., 2003). It is therefore of interest to investigate the coordinated morpho-physiological and biochemical responses of cluster root to P deficiency.

### 2.2 Root architecture

Root architecture refers to the overall spatial configuration of the root system, and it has significant effects on nutrient acquisition (Zhao et al., 2004; Koevoets et al., 2016; Li et al., 2016; Lynch, 2022). Root architecture displays high plasticity under P deficiency (Rellán-Álvarez et al., 2016; Amtmann and Shahzad, 2017; Motte et al., 2019). Modification of root architecture is the key strategy for plants to cope with low P stress and maximize Pi acquisition under P-limited conditions (Zhao et al., 2004; Li et al., 2016; Liu, 2021). Root architecture determines the distribution range and expansion degree of roots in soils, which is highly correlated with the P efficiency of legumes. The development of shallow root architecture is generally considered as an effective strategy for the legumes adaptation to P deficiency, such as common bean, mungbean (*Vigna radiata*) and soybean (Liao et al., 2004; Reddy et al., 2020; Seck et al., 2020). For example, in soybean, shallow root architecture and high lateral rooting are helpful traits in increasing Pi uptake compared to the deep root architecture. Furthermore, among an applied core collection of soybean, the cultivated soybean displays a shallow root architecture and high P efficiency, while the wild, climbing soybean exhibits a deep root architecture and low P efficiency (Zhao et al., 2004).

A range of quantitative trait loci (QTL) are reported to be related to root architecture and Pi acquisition (Liao et al., 2004). In legumes, for example, according to QTL analysis of basal root growth angle (BRGA) in bean recombinant inbred lines (RILs), the QTL for BRGA co-segregates with gain in yield under low P stress, and thus the BRGA has a major effect on PAE and yield under low-P conditions (Lynch and Brown, 2001; Liao et al., 2004). Furthermore, QTL analysis using bean RILs shows that some of the identified root traits, including those for BRGA, shallow basal root length and relative shallow basal root length, are associated with QTL for PAE (Liao et al., 2004). In addition, the QTL for basal root growth is also linked to the QTL for PAE in common bean (Beebe et al., 2006). Similarly, various QTL controlling root traits and P efficiency have been identified using soybean RILs; and the authors proposed that some of the identified QTL have great potential for genetic improvement of soybean with high P efficiency through a selection of root traits (Liang et al., 2010b).

Although key genes responsible for the QTL controlling root architecture in legumes have not been well characterized by forward genetic approaches, several genes possibly involved in modifying root architecture in response to Pi starvation have been identified by reverse genetics (Li et al., 2014; Yang et al., 2021a). For example, in soybean, GmEXPB2 is intrinsically involved in root system architecture responses to abiotic stresses, and overexpression of *GmEXPB2* modified soybean root architecture through expanding root hair density and size of the root hair zone (Guo et al., 2011; Li et al., 2015).

### 2.3 Induction of phosphate transporter genes

Phosphate transporters (PHTs) are known to control Pi uptake and transport in plants (Versaw and Garcia, 2017; Dai et al., 2022). PHTs have been identified in many plants and can be divided into four subfamilies (PHT1, PHT2, PHT3 and PHT4) according to their functional differences and subcellular localization. Among them, the PHT1 family is a multiprotein family that is localized in the plasma membrane for transporting Pi from apoplast to cytoplasm (Nussaume et al., 2011). Most *PHT1* genes are induced by Pi starvation in plant roots and are implicated in Pi uptake from soil or Pi translocation within plant tissues or cells (Liu et al., 2011; Remy et al., 2012). The role of PHT1 homologues in Pi uptake has been documented in legumes, such as *M. truncatula*, soybean and white lupin (Liu et al., 2001; Liu et al., 2008; Guo et al., 2022). Of the eleven *PHT1* homologues in *M. truncatula*, only *MtPT1*, *MtPT2*, *MtPT3* and *MtPT5* are induced by low P stress (Chiou et al., 2001; Liu et al., 2008). *MtPT1* is mainly expressed in the root and is likely involved in the Pi uptake (Chiou et al., 2001). *MtPT5* encodes a plasma membrane-localized Pi transporter (Liu et al., 2008). Ectopic expression of *MtPT5* complements the Pi uptake capability in the Arabidopsis *pht1;1pht1;4* double mutant, confirming its function in Pi uptake (Wang et al., 2022). In soybean, GmPT4 is a plasma membrane-localized Pi transporter; and overexpression of *GmPT4* increases the growth and Pi uptake in soybean, improving PAE (Guo et al., 2022). Pi-starvation-enhanced expression of *PHT1* genes are also found in white lupin and stylo (Liu et al., 2001; An et al., 2023). Overexpression of *SgPT1* from stylo can increase Pi uptake and enhance root growth in transgenic plants (An et al., 2023).

### 2.4 Root exudates

Insoluble P in soil can be divided into inorganic P and organic P. Inorganic P can be further classified into calcium phosphate (Ca-P), aluminum phosphate (Al-P) and iron phosphate (Fe-P), all of which is not easily available for the plant (Ao et al., 2014), while organic P mainly exists in the form of organic P esters and anhydrides, such as phytate, phospholipids, nucleotide and its derivatives (Simpson et al., 2011; Lorenzo-Orts et al., 2020).

Previous work indicates that the regulation of root organic acid exudation is an important process for inorganic P acquisition from the soil by chelating metal ions of insoluble phosphate, thereby increasing Pi concentration in soil solution (Peng et al., 2018; Lambers, 2022). Various types of organic acids secreted from roots are observed in legumes, such as white lupin (Lambers et al., 2013), soybean (Ryan et al., 2009), pigeon pea (*Cajanus cajan*) (Ishikawa et al., 2002) and common bean (Shen et al., 2002). For example, citrate and malate are the major organic acids secreted from cluster roots of white lupin under low P stress, which can help to increase Pi concentration in the rhizosphere (Vance, 2010; Lambers et al., 2013). Furthermore, organic acid exudation from a low-P-tolerant soybean genotype was higher than that of a low-P-sensitive soybean genotype (Zhang et al., 2011).

Organic acid synthesis and exudation are controlled by a variety of genes in legumes (Ryan et al., 2001; Wang et al., 2013; López-Arredondo et al., 2014). For example, organic acid synthesis is significantly increased in roots of transgenic alfalfa overexpressing the malate dehydrogenase gene, *MDH*, which is beneficial for increasing Pi uptake (Tesfaye et al., 2001; Tesfaye et al., 2003). Similar roles are observed in *GmMDH12* from soybean and *SgMDH1* from stylo (Chen et al., 2015; Zhu et al., 2021; Song et al., 2022). In addition, it has been reported that overexpression of citrate synthase (*DcCs*) from *Daucus carota* increases the synthesis and exudation of citrate in transgenic pigeonpea; and transgenic lines display enhancement of root growth compared to the wild type (Hussain et al., 2016). To date, the well-characterized malate and citrate transporters are members belonging to Aluminium Active Malate Transporter (ALMT) and Multidrug and Toxic Compound Extrusion (MATE) families, respectively (Sasaki et al., 2004; Hoekenga et al., 2006). For example, a group of *GmALMT* genes are regulated by P deficiency in soybean. Among them, GmALMT5, localized to the plasma membrane, is enhanced by Pi starvation in soybean roots; both root growth and P content of transgenic Arabidopsis overexpressing *GmALMT5* are increased when grown in a medium supplied with Ca-P as the external P source (Peng et al., 2018). Similarly, LaALMT1 is also characterized to be the plasma membrane-localized malate transporter in white lupin (Zhou et al., 2020). In addition, several genes encoding citrate transporters have also been reported in legumes, such as *VuMATE* in rice bean (*Vigna umbellata*) and *LaMATE1/3* in white lupin, all of which are induced by P deficiency (Wang et al., 2013; López-Arredondo et al., 2014; Zhou et al., 2021). For instance, *LaMATE1/3* exhibit the highest expression in mature cluster root under low-P conditions; mediating citrate transport when *LaMATE1/3* are expressed in oocytes, suggesting the role for *LaMATE1/3* in regulating citrate transport during low P stress (Zhou et al., 2021). In addition, secreting protons by legume root can acidify the rhizosphere soil, thereby improving the bioavailability of insoluble P (Kouas et al., 2009). For example, a plasma-membrane transporter GmVP2 has recently been reported to mediate H^+^ exudation from root of soybean exposed to low P treatment; overexpression of *GmVP2* in Arabidopsis can increase H^+^ exudation, promote root growth and increase Pi availability (Xie et al., 2022).

As mentioned before, about 30-65% of insoluble P in soil exists in the form of organic P, which can only be utilized by plants *via* the participation of various phosphoesterases, such as phosphatases, phosphodiesterases and nucleotidases (Matange et al., 2015; Tian and Liao, 2015; Yang et al., 2017). Purple acid phosphatases (PAPs) are among the most identified phosphoesterases in plants, which belong to the hydrolases that hydrolyze organic P to release inorganic Pi for plant uptake (Tian and Liao, 2015; Wu et al., 2018). It has been demonstrated that the root-associated/secreted PAPs are either associated with root surfaces or secreted into the rhizosphere, scavenging Pi from external organic P and increasing Pi availability (López-Arredondo et al., 2014; Tian and Liao, 2015; Wang and Liu, 2018). The Pi-starvation-increased activity of root-associated/secreted acid phosphatase is observed in common bean, stylo and peanut (*Arachis hypogaea*) (Liang et al., 2010a; Wei et al., 2022; Liu et al., 2016, 2018).

A set of PSI-secreted PAPs has been identified in legume plants, for example, LaSAP1/2 from white lupin (Wasaki et al., 2000; Wasaki et al., 2009), PvPAP3 from common bean (Liang et al., 2010a; Liang et al., 2012), and GmPAP7a/7b from soybean (Zhu et al., 2020). GmPAP14 is a secreted PAP in soybean. *GmPAP14* overexpression increases secreted APase and phytase activities, contributing to external phytate utilization and growth enhancement (Kong et al., 2018). Similar roles for GmPAP7a and GmPAP7b have been found in soybean and these two PAP members exhibit high activities against adenosine triphosphate (ATP) *in vitro*. Overexpression of *GmPAP7a* and *GmPAP7b* is able to increase root-associated APase activities, thereby improving utilization of organic P in soybean (Zhu et al., 2020). In common bean, PvPAP3 is found to localize to the plasma membrane and apoplast. The transcript of *PvPAP3* is induced by Pi starvation in roots of common bean, especially in the P-efficient genotype; and overexpression of *PvPAP3* increases the growth and P content in bean hairy roots and Arabidopsis when ATP and dNTPs are supplied as the sole external P source, respectively (Liang et al., 2010a; Liang et al., 2012). In addition, overexpression of *MtPHY1* or *MtPAP1* increases the ability of organic P utilization in both white clover (*Trifolium repens*) and alfalfa (Ma et al., 2009; Ma et al., 2012). The roles of other PAP homologues, such as SgPAP7/10/23/26 from stylo and CaPAP7 from chickpea in organic P utilization have also been elucidated (Liu et al., 2016; Bhadouria et al., 2017; Liu et al., 2018). These studies suggest that PSI-secreted PAPs play an important role in the utilization of external organic P for improved P acquisition.

### 2.5 Symbiotic association with soil microorganisms

**Figure 2:**
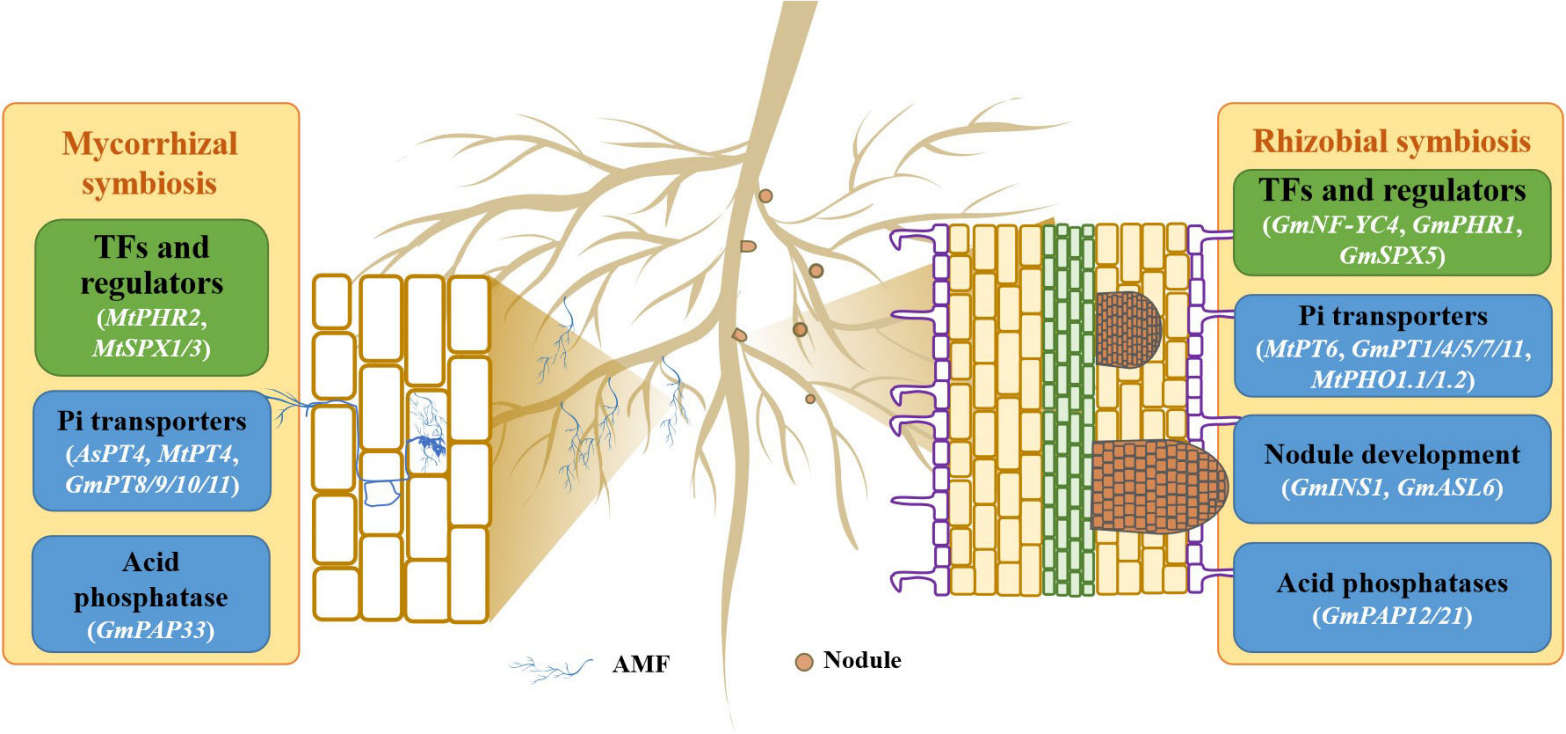
Genes involved in symbiotic interactions in the response of legumes to P deficiency. A model for mycorrhizal and rhizobial symbioses is presented. AMF, arbuscular mycorrhizal fungi.

In soils, plant roots can interact with many rhizosphere microorganisms, such as mycorrhizal fungi and phospho-relieving bacteria, regulating Pi uptake and its utilization (Smith et al., 2003; Smith et al., 2004; Liang et al., 2014). Most of the legumes can be infected by mycorrhizal fungi to form a symbiotic system. The formation of plant-mycorrhizal fungal symbiosis is one of the vital mechanisms of plant adaption to low P stress (Smith et al., 2003; Smith et al., 2004), which can improve plant P efficiency (**Figure 2**). In plant-mycorrhizal fungi symbiosis, mycorrhizal association generates a large number of extra-root hyphae which can extend beyond the P-deficient roots, but also enter into the small soil particle gaps to improve the spatial utilization of P in soils (Bago, 2000; Wang et al., 2011). Mycorrhizal fungi can promote Pi uptake and transport by regulating the expression of *PT* genes in the extracorporeal filaments and roots of the host plant. A set of *PT* homologues are induced by mycorrhizal fungi inoculation in host plants, such as *MtPT4/6* in *M. truncatula*, *AsPT4* in *Astragalus sinicu* and *GmPT8/9/10/11* in soybean (Harrison et al., 2002; Tamura et al., 2012; Xie et al., 2013; Wang et al., 2016; Cui et al., 2019). For example, *MtPT4* encoding a low-affinity Pi transporter is expressed in mycorrhizal roots of *M. truncatula*. Complemental analysis shows that MtPT4 can enhance Pi uptake in yeast (*Saccharomyces cerevisiae*) cells, suggesting that MtPT4 is involved in Pi acquisition from arbuscules in *M. truncatula* (Harrison et al., 2002; Volpe et al., 2016). In soybean, GmPT10 and GmPT11 are two mycorrhiza-inducible Pi transporters that can complement Pi transport in the yeast mutant PAM2, which is lacked PHO84 and PHO89, two high-affinity Pi transporters (Tamura et al., 2012). In addition, *PAP* genes have also been characterized to involve in plant-mycorrhizal fungi symbiosis (Li et al., 2017; Li et al., 2019; Wang et al., 2020). The plasma membrane-localized GmPAP33 is found to be mainly expressed in arbuscule-containing cells; overexpression of *GmPAP33* significantly increases the percentages of large arbuscules and P content of transgenic soybean when inoculated with AM fungi (Li et al., 2019).

On the other hand, legumes are able to develop symbioses with rhizobia to generate a special organ, nodule, which can release an abundance of protons to acidify the rhizosphere, thereby increasing Pi availability in soils (Qin et al., 2011; Ding et al., 2012). It has been reported that the growth and N and P contents are increased in soybean after inoculating with rhizobia in low-P acid soils (Cheng et al., 2009). A variety of PSI genes and proteins have been identified in nodules exposed to P deficiency (**Figure 2**; Hernández et al., 2009; Chen et al., 2011; Cabeza et al., 2014; Xue et al., 2018; Zhong et al., 2023). For example, 1140 and 2055 genes have been found to be regulated by P deficiency in nodules of *M. truncatula* and soybean, respectively (Cabeza et al., 2014; Xue et al., 2018). Among them, a group of Pi transporter genes have been reported to be involved in improving Pi uptake in nodules. For example, *GmPT7*, a nodule-localized Pi transporter, is induced by low P stress in soybean nodules. *GmPT7* is found to be mainly expressed in the outer cortex and N fixing zones of the nodules; overexpression of *GmPT7* in soybean increased nodulation, P content and soybean yield, suggesting that GmPT7 is responsible for direct Pi entry to nodules (Chen et al., 2019). Unlike GmPT7, GmPT5 is proposed to function in transporting Pi from roots to nodules, thereby regulating nodulation and soybean growth (Qin et al., 2012). In addition, *GmPT1/4/11* are also implicated in Pi homeostasis and nodulation in soybean (Qin et al., 2012; Chen et al., 2019; Lu et al., 2020). In *M. truncatula*, *MtPT6* is preferentially expressed in vascular bundles, cortical cells, and fixation zone cells of nodules. Functional analysis confirms that MtPT6 is a typical Pi transporter and can increase PAE in plants (Cao et al., 2021). Furthermore, *MtPHO1.1* and *MtPHO1.2* are expressed not only across the various nodule zones but also the root vascular system, participating in transporting Pi from infected nodule cells to bacteroids in *M. truncatula* (Müller, 2021; Nguyen et al., 2021). In addition, the transcript of *GmPAP12* is gradually increased during soybean nodule growth. Subsequent analysis shows that overexpression of *GmPAP12* leads to increase nodule number, shoot dry weight, and N and P content of transgenic composite soybean plant under low P stress, suggesting the roles of *GmPAP12* involved in nodulation and SNF in soybean (Wang et al., 2020). Among the nine β-expansin members, the expression of *GmINS1* is found to be enhanced by Pi starvation in soybean nodule; overexpression of *GmINS1* increases nodule size and N_2_ fixation capacity, ultimately enhancing P content, plant biomass and soybean yield (Li et al., 2018; Yang et al., 2021b). Although only a few genes involved in symbiosis have been identified, their function highlights the importance of symbiosis for improving legume adaption to low P stress.

### 2.6 Regulation of Pi signaling network

Physiological and biochemical changes of plants in response to low P stress are mediated by a complex signaling network, including local and systemic sensing and signaling. A set of important components shown in **Figure 1**, such as transcription factors and regulators, have been demonstrated to be involved in the Pi signaling network. Among them, an R2R3 MYB member, Phosphate Starvation Response 1 (PHR1) is the most well-characterized transcription factor involved in Pi starvation responses in the Pi signaling network (Chiou and Lin, 2011). PHR1 or PHR1-like is considered as the central transcription factor of Pi signaling network through the regulation of a lot of downstream PSI genes (Wang et al., 2009; Bustos et al., 2010; Wang et al., 2014; Zhong et al., 2018). To date, several *PHR1* genes have been identified in legumes, such as *GmPHR1/4/25* from soybean (Xue et al., 2017; Lu et al., 2020) and *PvPHR1* from common bean (Valdés-López et al., 2008). For example, in soybean, the Pi starvation up-regulated gene *GmPHR25* is an important regulator in the Pi signaling network that controls Pi homeostasis in soybean; and overexpressing *GmPHR25* results in increasing Pi concentration in transgenic soybean hairy roots, probably through enhancing the expression of several high-affinity Pi transporters (*GmPTs*) and *GmPAP14/21* (Xue et al., 2017). In common bean, PvPHR1 is a positive regulator of the Pi signaling network. The transcript of *PvPHR1* is increased by Pi starvation in both leaves and roots; and the knockdown of *PvPHR1* leads to a decreased expression of a set of PSI genes, such as *PvPHT1*, *PvPHO1*, *Pv4*, *PvRNS* and *PvAPC5*, thereby decreasing Pi concentrations in composite common bean plants (Valdés-López et al., 2008).

In addition, SPX domain-containing proteins also have been characterized as key sensors and regulators of Pi homeostasis and signaling in soybean, common bean and *M. truncatula* (Yao et al., 2014a; Yao et al., 2014b; Wang et al., 2021; Zhuang et al., 2021). For instance, in *M. truncatula*, two SPX domain-containing proteins, MtSPX1 and MtSPX3, are found to be localized in the cytoplasm and the nucleus and can also interact with MtPHR2. The expressions of *MtSPX1* and *MtSPX3* are increased by Pi starvation in arbuscule-containing cells. Lost function of *MtSPX1* and *MtSPX3* results in decreased root colonization and arbuscule abundance as well as the expression of Rhizophagus Irregularis Elongation Factor (*RiEF*) and *MtPT4*, suggesting that *MtSPX1* and *MtSPX3* play important roles in maintaining arbuscular mycorrhizal symbiosis in *M. truncatula* (Wang et al., 2021). In common bean, PvSPX1 is a positive regulator in the Pi signaling network. PvSPX1 can regulate a set of downstream of PSI genes that are involved in Pi transport, translocation and homeostasis, such as *PvPAP1/2/3/4/5*, *PvPS2:1*, *Pv4* and *PvPHR1* (Yao et al., 2014b). A similar role of GmSPX3 has been implicated in soybean (Yao et al., 2014a). In addition to interaction with PHR, one of the GmSPX members in soybean, GmSPX5, has been reported to interact with the transcription factor GmNF-YC4 to activate *GmASL6* expression, mediating nodule development through regulating asparagine metabolic processes (Zhuang et al., 2021).

Other transcription factors belonging to bHLH, WRKY, SPL, EIN3, ZAT and GARP families are also involved in the regulation of PSI genes in the Pi signaling network (Zhang et al., 2014; Scheible and Rojas-Triana, 2015; Song and Liu, 2015). For example, in soybean, GmPTF1 is an HLH transcription factor responsible for activation of GmEXPB2, modifying root architecture during P deficiency (Li et al., 2014; Yang et al., 2021a). In addition, other regulators, such as miRNAs and PHO2, have also been reported to be involved in the Pi-starvation response in many legume plants, such as common bean (Valdés-López et al., 2010), white lupin (Zhu et al., 2010), soybean (Zeng et al., 2010), *M. truncatula* (Boualem et al., 2008) and alfalfa (Li et al., 2018). For example, *PvPHO2*, encoding E2 ubiquitin ligase, is a target that is negatively regulated by *miR399* in common bean; *PvPHO2* is suppressed by P deficiency and it negatively regulates PSI genes, including *PvAPC5*, *PvAP* and *PvPHT1* (Valdés-López et al., 2008; Ramírez et al., 2013).

## 3 Strategies for improving PAE of legumes

Due to the non-renewable, limited P resources and severe environmental problems associated with excess P mining and fertilizer application, more attention has been paid to improve PAE in legumes for sustainable agriculture. As discussed before, optimizing root responses is a key strategy for improving PAE and plant tolerance to low P stress. Strategies for improving PAE of legumes can be achieved by conventional breeding, marker-assisted selection (MAS) breeding or genetic engineering with target gene transformation (Yan et al., 2004; Ojeda-Rivera et al., 2022).

As conventional breeding for improved crop P efficiency is mainly based on phenotypic selection for root systems which is difficult and time-consuming, another important approach for improving plant PAE of crops in modern agriculture is based on the identification of QTL for the trait of interest with molecular markers. A pioneering work in rice has demonstrated that Pi uptake 1 (*Pup1*) is a key QTL for improving Pi uptake (Gamuyao et al., 2012). The P efficiency-related gene, phosphorus-starvation tolerance 1 (*PSTOL1*), responsible for the *Pup1* QTL, acts as an enhancer of root growth and Pi uptake in rice and Arabidopsis (Wissuwa et al., 1998; Wissuwa et al., 2002; Chin et al., 2011; Gamuyao et al., 2012; Neelam et al., 2017), suggesting the potential use of *Pup1* or *PSTOL1* in breeding crop varieties with high P efficiency. In addition, it has been demonstrated that *SbPSTOL1*, the homologue of *OsPSTOL1* in sorghum (*Sorghum bicolor*), not only involves in enhancing root surface area but also improving root system architecture, contributing to increase in grain yield under low-P condition (Hufnagel et al., 2014; Leiser et al., 2014). In common bean, QTL analysis was performed using RILs population for the root traits, including root-hair density, root-hair length, H^+^ exudation and total acid exudation, which shows that QTL for H^+^ exudation and total acid exudation are closely linked with QTL for Pi uptake; and the authors suggest that molecular markers linked to the target root traits might be potentially used for screening of phenotypic root traits that contribute to improved P efficiency in the breeding process (Yan et al., 2004). In addition, several QTL for root traits and P efficiency have also been identified in soybean (Liang et al., 2010b; Yang et al., 2019; Seck et al., 2020) and other legumes, such as chickpea (Varshney et al., 2019). However, the achievements in identifying QTL for P efficiency are still limited due to the epistatic effects and their interactions with the environment, and most QTL regions are needed to be narrowed down so that only target genes are identified. In addition, genome-wide association study (GWAS) of root traits associated with P efficiency could also provide a helpful solution to identify key genes and their interactions in crops. A recent study has shown that a genetic locus, component of phosphorus uptake 1 (*CPU1*) contributes to P efficiency based on GWAS of PAE in a soybean core collection (Guo et al., 2022). Furthermore, a SEC12-like gene, *GmPHF1*, identified as the causal gene for CPU1, is proven to mediate Pi uptake in soybean (Guo et al., 2022).

With the rapid development of transgenic techniques, numerous genes have been successfully introduced into different legumes to improve PAE, which are summarized in **Table 1**. For example, in soybean, GmPT4 is a plasma membrane-localized Pi transporter and *GmPT4*-overexpression in soybean plants displays higher PAE and biomass than the wild type (Guo et al., 2022). Furthermore, transgenic soybean plants overexpressing *GmEXPB2* exhibit an increase in root length and root hair density, resulting in an increase of PAE in soybean (Zhou et al., 2014; Li et al., 2015; Yang et al., 2021a). GmPAP7a and GmPAP7b possess high activities against ATP *in vitro*. Overexpression of *GmPAP7a* and *GmPAP7b* improves the utilization of organic P in soybean (Zhu et al., 2020). In addition, overexpression of *GmETO1* can increase PAE in plants and tolerance to low P stress (Zhang et al., 2020). Overexpression of *PvPAP3* increases the growth and P content in bean hairy roots when supplied with organic P (Liang et al., 2010a; Liang et al., 2012). Similarly, both *MtPHY1* and *MtPAP1* transgenic white clover and alfalfa plants display high PAE and tolerance to low P (Ma et al., 2009; Ma et al., 2012). Genes related to organic acid synthesis, such as *MDH* in alfalfa and *DcCs* in *Daucus carota* can also improve Pi uptake in transgenic plants (Tesfaye et al., 2001; Tesfaye et al., 2003; Hussain et al., 2016). Although none of the transgenic plants generated with high PAE have been released for commercial use, transgenic techniques are shown to be effective in improving plant PAE. Such improvements are essential at least to adjust how we manage crop yield and excess P fertilizer inputs.

**Table 1:** Genes that have been used in transgenic modification for improving P acquisition efficiency in legumes.

Gene name	Description	Species	Transformed plant species	Tissue expression	Main function	References
*GmEXPB2*	β-expansin protein	*Glycine max*	*Glycine max*	Root tip	Increased root growth and Pi acquisition	Guo et al., 2011; Zhou et al., 2014; Li et al., 2015; Yang et al., 2021a
*SgEXPB1*	β-expansin protein	*Stylosanthes guianensis*	*Stylosanthes guianensis*	Root, Seed	Increased root growth and Pi acquisition	Wang et al., 2023
*GmEXLB1*	expansin-like B protein	*Glycine max*	*Arabidopsis thaliana*	Root	Increased root growth and Pi acquisition	Kong et al., 2019
*GmPT4*	Phosphate transporter	*Glycine max*	*Glycine max*	Root	Increased plant growth and Pi uptake	Guo et al., 2022
*GmPT7*	Phosphate transporter	*Glycine max*	*Glycine max*	Nodule	Increased nodulation, Pi uptake and plant biomass	Chen et al., 2019
*MtPT4*	Phosphate transporter	*Medicago truncatula*	*Medicago truncatula*	Mycorrhizal root	Relative to symbiotic Pi acquisition and AM symbiosis	Javot et al., 2007
*MtPT6*	Phosphate transporter	*Medicago truncatula*	*Medicago truncatula*	Root, Nodule	Increased plant growth and Pi uptake	Cao et al., 2021
*SgPT1*	Phosphate transporter	*Stylosanthes guianensis*	*Arabidopsis thaliana*	Root, Stem, Leaf	Increased plant growth and Pi uptake	An et al., 2023
*AfPhyA*	Phytase	*Aspergillus ficuum*	*Glycine max*	–	Increased phytase activity and Pi uptake	Li et al., 2009
*MtPHY1*	Phytase	*Medicago truncatula*	*Trifolium repens, Medicago sativa*	–	Improved utilization of organic P and plant biomass	Ma et al., 2009; Ma et al., 2012
*SgPAP23*	Phytase	*Stylosanthes guianensis*	*Arabidopsis thaliana Phaseolus vulgaris*	–	Improved utilization of organic P and plant biomass	Liu et al., 2018
*GmPAP7a*	Purple acid phosphatase	*Glycine max*	*Glycine max*	Root	Improved utilization of organic P and plant biomass	Zhu et al., 2020
*GmPAP7b*	Purple acid phosphatase	*Glycine max*	*Glycine max*	Root	Improved utilization of organic P and plant biomass	Zhu et al., 2020
*MtPAP1*	Purple acid phosphatase	*Medicago truncatula*	*Trifolium repens Medicago sativa*	–	Improved utilization of organic P and plant biomass	Ma et al., 2009; Ma et al., 2012
*PvPAP3*	Purple acid phosphatase	*Phaseolus vulgaris*	*Phaseolus vulgaris*	Root	Increased root hair density and uptake of extracellular organic P	Liang et al., 2010a, Liang et al., 2012
*SgPAP7*	Purple acid phosphatase	*Stylosanthes guianensis*	*Phaseolus vulgaris*	Shoot, Root	Improved utilization of organic P and plant biomass	Liu et al., 2016
*SgPAP10*	Purple acid phosphatase	*Stylosanthes guianensis*	*Phaseolus vulgaris*	Shoot, Root	Improved utilization of organic P and plant biomass	Liu et al., 2016
*SgPAP26*	Purple acid phosphatase	*Stylosanthes guianensis*	*Phaseolus vulgaris*	Root	Improved utilization of organic P and plant biomass	Liu et al., 2016
*LASAP2*	Acid phosphatase	*Lupinus albus*	*Tobacco*	Cluster root	Improved Pi mobilization and uptake	Wasaki et al., 2009
*GmALMT5*	Aluminum active malate transporter	*Glycine max*	*Arabidopsis thaliana*	Root	Increased organic acid exudation and Pi acquisition	Peng et al., 2018
*DcCs*	Citrate synthase	*Daucus carota*	*Cajanus cajan*	–	Improved Pi mobilization and uptake	Hussain et al., 2016
*GmVP2*	H^+^-pyrophosphatase	*Glycine max*	*Arabidopsis thaliana*	Root	Increased root H^+^ exudation, root growth and Pi availability	Xie et al., 2022
*GmSPX3*	SPX domain-containing proteins	*Glycine max*	*Glycine max*	Root	Increased plant growth and Pi acquisition	Yao et al., 2014a
*Gm6PGDH1*	6-phosphogluconate dehydrogenase	*Glycine max*	*Glycine max*	Root, Flower	Increased root growth and Pi acquisition	Li et al., 2021
*GmETO1*	Ethylene-overproduction protein	*Glycine max*	*Glycine max*	Root	Increased root growth and Pi acquisition	Zhang et al., 2020
*GmPHF1*	Phosphate transporter traffic facilitator	*Glycine max*	*Glycine max*	Root	Increased plant growth and Pi uptake	Guo et al., 2022
*GmWRKY45*	WRKY transcript factor	*Glycine max*	*Arabidopsis thaliana*	Root	Increased lateral root growth and Pi uptake	Li et al., 2020
*GmWRKY46*	WRKY transcript factor	*Glycine max*	*Arabidopsis thaliana*	Root	Increased root growth and Pi uptake	Li et al., 2021

Modified from Ramaekers et al. (2010) and Zhang et al. (2014). ‘-’ means not detected.

In addition to molecular breeding and genetic engineering, the inoculation of crops with plant growth promoting rhizobacteria (PGPR) is one of the most effective strategies for improving the PAE of legumes, such as soybean, common bean, and chickpea (Pérez-Montaño et al., 2014; Mohamed et al., 2019; Lazali and Drevon, 2021). PGPR are free-living bacteria from the genera *Pseudomonas* and *Bacillus* that can colonize plant roots, playing crucial roles in plant growth and development. The PGPR can promote plant growth by producing phytohormones, enhancing root development, solubilizing Pi, thereby increasing PAE in plants (Mantelin and Touraine, 2004; Yang et al., 2009). For example, *Bacillus subtilis* and *Pseudomonas fluorescence* are regarded as the bio-fertilizers for plants; after inoculation of these bacteria, the yield and Pi uptake are significantly increased in common bean (Mohamed et al., 2019). In chickpea, application of PGPR can enhance plant growth and PAE by increasing exudation of organic acids from roots (Israr et al., 2016). In addition, Pi-solubilizing bacteria are vital components of PGPR for legumes to acquire more Pi from soil (Rosas et al., 2006; Tabassum et al., 2017). For example, inoculating Pi-solubilizing bacteria, such as *Bradyrhizobium japonicum* and *Pseudomonas putida*, can contribute to improve the shoot and root growth of soybean by solubilizing Pi from the tricalcium phosphate (Rosas et al., 2006). Therefore, application of PGPR is a useful strategy for improving PAE of legumes.

## 4 Limitations and future perspectives

P is a major nutrient that is essential for crop growth performance and productivity. As P availability in most agricultural soils is low, inorganic P fertilizers are overused in intensive cropping systems to ensure crop production stability, leading to negative environmental impacts. At the same time, the low availability of P in soils and insufficient financial support to access P fertilizers in some developing countries keep many smallholder farmers from growing crops with high productivity and quality. Legumes possess numerous economic and environmental benefits in agricultural systems, but their productivity is severely affected by low P availability in soil. Thus, developing P-efficient legume cultivars with high yields using less P fertilizer inputs could contribute not only to sustainable and regenerative agriculture but also to global food and nutritional security.

As discussed in this review, enhanced PAE through modification of root growth and response is a key strategy for increasing plant P efficiency under limited P supply. To date, numerous PSI genes have been shown to be associated with increasing PAE, such as genes that are involved in root growth, Pi uptake, and Pi signaling network. Despite this significant progress in recent years, the current understanding of the specific mechanisms and regulatory aspects of controlling root growth response to P deficiency in legumes is incomplete. Furthermore, it is not easy to develop legume or even other crop cultivars with desirable root traits by manipulating a single gene without a trade-off in carbon cost and crop productivity. These are some of the aspects that need to be considered in improving PAE of legume crops, and this will require enhanced collaboration between plant breeders, molecular biologists and plant physiologists.

With the advance in large-scale omics approaches, the application of the reliable and accurate next-generation sequencing (NGS) and transcriptomic technologies, as well as in-depth studies on post-transcriptional regulation can accelerate genetic improvement of crops that are able to cope with low P stress by identifying novel functional and regulatory genes related to P efficiency. In addition, QTL analysis and GWAS of root traits with P efficiency are helpful for identifying candidate genes in crops. Therefore, making full use of the advantages of these new approaches might help to develop high P-efficient legume cultivars more quickly in the near future. Furthermore, in order to fully understand the mechanisms underlying plant cell response to P deficiency, single-cell RNA sequencing can be applied to investigate the dynamic responses of plant cells to Pi starvation.

Although plants display root plasticity during P deficiency with improved root growth and thus increasing PAE, this active response of root might occur at a certain P level and can not be maintained during all growth stages of the plant, especially under severe P deficiency. When Pi uptake reaches its maximum level, modification of PUE is an important complementary strategy that is also needed for improving P efficiency in plants. A higher PUE can be achieved through efficient re-translocation, re-distribution and re-use of Pi from organic P pools in cells or tissues of plants, which involves various transport and metabolic processes, thereby reducing P depletion from soils as well as the dependence of smallholder farmers on P fertilizer inputs. Thus, additional research efforts are needed to combine PAE with PUE in legumes to improve their adaptation to low P soils.

To unravel the key mechanisms responsible for complex interactions among plant root systems, rhizosphere and P status in soils (e.g., interactions of root exudates with microbes and soil chemistry to facilitate P mobilization), transdisciplinary research efforts are needed that will require expertise in plant biology, soil science, rhizosphere biology and ecophysiology. Furthermore, for improving P efficiency of legume crops grown in low P soils, basic and applied research work is needed to find the perfect breeding approach that incorporates SNF capability, adaptation to climate variability and change, mono- and intercropping cultivation models, and agronomic management conditions. In addition, some legume plants are able to acquire less available forms of soil P and these legume genotypes could become major focus for future investigation on gene identification and gene transfer.

## Author contributions

ZC and YL conceptualized the manuscript. ZC, LW, SZ and YL wrote the original draft. JC, IR, GL, ZC and YL reviewed and edited the manuscript. All authors contributed to the article and approved the submitted version.
